# Literary Notes

**Published:** 1905-09

**Authors:** 


### LITERARY NOTES.

John Lane Company, The Bodley Head, London and New York, has begun to issue a series of medical and surgical handbooks under the title The practitioner's handbooks. They are written by specialists for the use of general practitioners and are under the general editorship of Harry Roberts. The first volume of the series is devoted to "The Rheumatic Diseases" and has been entrusted to J. O. Symes, assistant physician and bacteriologist to the Bristol General Hospital. The second is concerned with "Hysteria and Neurasthenia," and is the work of J. Michell Clarke, physician to Bristol General Hospital and professor of pathology, University College, Bristol.

---

Messrs. Lea Brothers & Company, Philadelphia, announce that the National Standard Dispensatory, by Hare, Caspari and Rusby, will be ready for sale September 1, the date when the new U. S. Pharmacopeia goes into effect. By authority of the convention it will contain every article in the new U. S. P., as well as the explanations and instructions necessary to understand and apply the brief statements to which the official guide is restricted.

---

The F. A. Davis Company, Philadelphia, announce the early publication of a Treatise on the motor apparatus of the eyes, embracing an exposition of the anomalies of the ocular adjustments and their treatment, with the anatomy and physiology of the eye muscles and their accessories, by Dr. George T. Stevens, of New York.

---

Messrs. P. Blakiston's Son & Company, Philadelphia, announce the forthcoming publication of A manual and atlas of orthopedic surgery by Dr. James K. Young, professor of orthopedic surgery, Philadelphia Polyclinic.

